# High-Efficiency SERS of 4-Mercaptobenzoic Acid and Biphenyl-4,4′-Dithiol via Nanoparticle-on-Mirror Plasmonic Nanocavities

**DOI:** 10.3390/nano15060421

**Published:** 2025-03-09

**Authors:** Wangze Li, Yifan Zhu, Jinze Li, Lei Guo, Xilin Zhou, Xin Xie, Zhengkun Fu, Huan Chen, Hairong Zheng

**Affiliations:** 1 School of Physics and Information Technology, Shaanxi Normal University, Xi’an 710119, China; wzli@snnu.edu.cn (W.L.); 2022300952@snnu.edu.cn (Y.Z.); 2023303956@snnu.edu.cn (J.L.); glei@snnu.edu.cn (L.G.); xilin@snnu.edu.cn (X.Z.); xinxie@snnu.edu.cn (X.X.); zkfu@snnu.edu.cn (Z.F.); 2 Xi’an Key Laboratory of Optical Information Manipulation and Augmentation, Xi’an 710062, China

**Keywords:** surface enhanced Raman scattering, plasmonic nanocavities, monomolecular layer

## Abstract

Surface-enhanced Raman scattering (SERS) technology has important applications in many fields, such as biomedicine, environmental monitoring, and food safety. Plasmonic nanocavities have the ability to superdiffract localized light and enhance light-matter interactions. As a key SERS active substrate, research on plasmonic nanocavities has made significant progress regarding the enhancement mechanism, the utilization of hotspots for the detection of specific molecular groups, and practical applications. However, challenges related to improving the enhancement factor of nanocavity SERS, enhancing the stability and reproducibility of hotspots, and enabling the detection of single-molecule layers remain. In this study, we adopt a bottom-up approach to construct a silver microplate–molecule–multi-sized silver nanosphere nanoparticle-on-mirror (NPoM) nanocavity and achieve the efficient stable enhancement of Raman scattering from 4-mercaptobenzoic acid and biphenyl-4,4′-dithiol molecules via the electromagnetic mechanism. By characterizing the fabricated nanocavity using dark-field scattering and micro-confocal Raman scattering, we observed that the Raman scattering intensity in the NPoM nanocavity was enhanced by a factor of 10^3^ compared to that of individual silver nanospheres. Furthermore, we achieved the efficient stabilization of SERS by precisely tuning the size of the silver nanospheres to match their resonance frequency with the Raman shift of the target molecules. This approach offers a valuable reference for the detection of various single-molecule layers and demonstrates significant potential for applications in biosensing and chemical analysis.

## 1. Introduction

Surface plasmons are surface-bound electromagnetic waves formed by the collective coherent oscillations of free electrons at metal–dielectric interfaces or nanostructures under the action of an external light field [1]. When the frequency of the incident light matches the frequency of the collective oscillations of the electrons, the incident light can interact resonantly with the electrons on the metal surface, and the strength of the electromagnetic field around the metallic nanomaterials is significantly enhanced [2]. In recent years, the surface plasmon resonance of metal nanoparticles has been utilized to achieve site-specific Raman enhancement and Raman imaging [3] inside biomolecules and cells, which is widely applied in nanoelectronics, diagnostics, molecular imaging and biomedicine [4,5]. Among these, the enhancement effect of silver nanospheres on molecular Raman scattering has attracted much attention. From the perspective of the enhancement effect in Surface-Enhanced Raman Spectroscopy (SERS), silver nanospheres exhibit a strong electromagnetic enhancement, which significantly boosts the detection sensitivity, enabling single-molecule detection [6]. Additionally, these nanospheres possess rich plasmonic properties, enabling the precise tuning of their characteristics. By adjusting factors such as the size, shape, and surrounding environment, the resonance frequency of the silver nanospheres can be optimized to match the specific Raman shift enhancement required for improved performance [7]. In terms of chemical properties, although silver nanospheres have relatively poor stability, they can maintain a stable performance in different environments and ensure signal reproducibility after surface modification or coating with an inert shell [8]. Additionally, surface modification can reduce the toxicity of silver nanoparticles [9], making them suitable for biomedical applications [10]. Moreover, they have strong modifiability and can be connected with specific recognition molecules or groups, which is used to construct high-performance sensors and improve the selectivity and accuracy of detection.

Typically, the molecules detected by SERS are adsorbed within silver nanosphere aggregates or plasmonic nanostructures [11,12]. Among them, the SERS effect has been the main focus of many experimental and theoretical studies [13], which can enhance Raman scattering by a factor of 10^8^ to 10^12^ [14] and even detect individual molecules [15,16]. In plasmonic nanostructures, metallic nanocavities utilize localized surface plasmon resonance (LSPR) to confine the electric field within nanostructures whose radii are much smaller than the wavelength [17]. They possess the ability to localize light beyond the diffraction limit and provide an extremely small cavity mode volume. This is extremely beneficial for exploring the limits of the interaction between light and matter at the nanoscale and lays a theoretical foundation for the development of SERS technology [18].

On-chip plasmonic nanocavity, also known as the nanopatch optical antenna, NPoM nanocavity [19], and metal–insulator–metal (MIM) waveguide, is a powerful nano-optical manipulation platform widely used in nanoscale light–matter interaction studies [20]. NPoM nanocavity can realize the enhancement of Raman scattering through the electromagnetic mechanism (EM) [21], because the frequency of the incident light wave resonates with the characteristic frequency of the free electrons in the metal nanoparticles on the substrate, and subsequently drives the electrons to move with the amplitude of the optical field. During this process, the electromagnetic field on the surface of the substrate is enhanced, which strengthens the polarization of molecules and increases the dipole moments. Consequently, stronger Raman scattering is generated [22]. Meanwhile, a charge transfer phenomenon occurs between the SERS substrate and the molecules adsorbed on its surface. When electrons are transferred from the surface of the SERS substrate to the adsorbed molecules, metal–molecule bonds are formed, enhancing the Raman scattering [23]. The SERS technique based on NPoM nanocavities shows a wide range of applications in the fields of molecular detection and nanophononics [24], which can be used to study the surface properties and molecular structure of materials and help to understand the properties and applications of materials [25]. However, in practical applications, the stability and reproducibility of SERS enhancement effects can vary under different experimental conditions [26]. Therefore, further studies and improvements in enhancement stability, molecular specificity, background suppression, and multi-molecule detection capabilities are needed [27]. By exploring suitable molecules for the nanocavities and regulating modification methods, the high efficiency and stable enhancement of specific molecules can be improved, and the continuous background interference can be reduced [28].

In this paper, the highly efficient SERS detection of 4-mercaptobenzoic acid (4-MBA) and biphenyl-4,4′-dithiol (BPDT) molecules is achieved using a silver microplate–molecule–multi-sized silver nanosphere NPoM nanocavity. By modulating the size of the silver nanospheres used to construct the NPoM nanocavity and adjusting the interstitial molecular dimensions [29], and by combining single-particle dark-field scattering technology and in situ morphological characterization, the precise control and positioning of the resonance peaks in different NPoM nanocavities is achieved. By accurately monitoring the Raman scattering from molecules within individual NPoM nanocavities, it was found that the NPoM nanocavity can achieve up to 10^3^-fold signal enhancement when detecting specific molecular monolayers, such as 4-MBA and BPDT. This research demonstrates the potential application of the NPoM nanocavity, composed of silver microflakes, molecules and a multi-sized silver nanosphere, in biosensing and chemical analysis. It provides a theoretical basis and practical guidance for effectively suppressing continuous background interference and improving the sensitivity of SERS sensing by regulating the geometry of plasmonic nanocavities in future studies.

## 2. Materials and Methods

The atomically leveled monocrystalline silver micrometer flakes were prepared utilizing a wet chemical method. First, 10 mg of hydrazine sulfate and 10 mg of metol (4-Methylaminophenol sulfate) were dissolved in 10 mL of deionized water. Subsequently, 1 mg of silver nitrate (AgNO_3_) was dissolved in 10 mL of deionized water. Then, the previously prepared hydrazine sulfate–Metol mixed solution and the silver nitrate solution were slowly added dropwise to the reaction container in a 5:4 ratio. After the reaction, the precipitate was washed with an alcoholic solution to obtain clean single-crystalline silver micrometer plates (Ag MPs).

Single-crystal silver nanospheres of different sizes were synthesized by a chemical reduction method, which is one of the methods most widely used to synthesize silver nanospheres. The specific steps are as follows: silver nitrate was dissolved in water to prepare an aqueous silver nitrate solution with a concentration of 1 wt%. Subsequently, 2 mL of aqueous silver nitrate solution was added to 800 μL of ammonia (25%~28%), which formed a complex with the silver precursor and, at the same time, could buffer the pH of the reaction medium. Then, 2000 μL of Ag seed was dissolved into 4.75 mL of deionized water with thorough stirring. Subsequently, an aqueous solution of silver–ammonia complex (70 μL, 43 mM) and an aqueous solution of acrylic acid (AA) (2 mL, 2.9 mM) were sequentially added to the silver seed solution. After stirring for 1 h, pure 60 nm silver nanospheres were obtained by centrifugal washing with the addition of deionized water. In addition, the amount of Ag seed was adjusted to prepare quasi-spherical silver nanospheres with different sizes, and the sizes of the silver nanospheres obtained were 80 nm, 100 nm, and 120 nm when the amounts of Ag seed were 1000 μL, 400 μL, and 200 μL, respectively.

For the preparation of silver nanospheres coated with a monolayer and the NPoM nanocavity, 500 μL of silver nanospheres of different sizes, respectively, was added to 0.1 mM of 4-MBA and BPDT solutions. The mixtures were stirred at room temperature for 24 h. Then, deionized water was added, washed and centrifuged to obtain silver nanospheres coated with a monolayer. In this paper, a bottom-up method was adopted to construct the on-chip plasmonic nanocavities of the NPoM. Firstly, a diluted solution of single-crystalline silver micro-triangles was deposited on a silicon wafer. A solution of silver nanospheres, coated with a monolayer, was then spin-coated onto the substrate. The spin-coating process was performed at 250 revolutions per minute (RPM) for 15 s, followed by 1000 RPM for 60 s to facilitate the assembly of the nanospheres onto the single-crystalline silver microflakes. Afterward, the assembled samples were dried at 50 °C for 10 min, resulting in the formation of the on-chip plasmonic nanocavity structure.

The structural and morphological characterization of the samples involved in this study was mainly achieved using a scanning electron microscope (SEM, FEI-Nova Nano 450, Thermo Fisher Scientific, Waltham, MA, USA), with the surface morphology of the samples typically characterized at an acceleration voltage of 17.5 kV.

To evaluate the variation in the resonance wavelengths of nanocavities formed by silver nanospheres of different sizes, this study employed atomic layer deposition (ALD) to introduce Al_2_O_3_ isolation layers of varying thicknesses between the nanocavity gaps. First, several silicon substrates with pre-deposited silver microplates were prepared and placed into the ALD system, where the deposition process was carried out according to a preset program. Each deposition cycle added approximately 0.11 nm in thickness, continuing until the maximum deposition thickness reached 0.99 nm, which corresponds to the diameter of a single monolayer of 4-MBA or BPDT molecules. Different sizes of silver nanospheres were then added to each sample. Finally, these nanocavity samples with varying nanosphere sizes were tested under dark-field microscopy.

The setup for performing grazing incidence dark-field scattering measurements is shown in Figure S1. A halogen tungsten lamp was used to emit wide-field light in the range of 400–1000 nm, which was directed onto the sample at an angle of 30 degrees relative to the substrate. The scattered light from the sample was then collected through a 50× objective lens (NA = 0.5) and directed into an optical camera, allowing us to observe the scattering spots of various colors emitted from multiple nanocavities. By positioning the target nanocavity within the collection range and directing its scattered light into a spectrometer (Horiba, LabRAM HR Evolution, Kyoto, Japan), the dark-field scattering spectrum of a single nanocavity could be obtained. Raman spectroscopy measurements were carried out using a micro-confocal Raman system, as shown in the schematic diagram of the optical path in Figure S2. Depending on the different structural systems, the experimental conditions and measurement methods need to be optimized or adjusted. In this study, a 532 nm continuous laser with a power density of 1.26 kW/cm^2^ was used as the excitation source at room temperature, and the sample was focused and observed using a 50× objective lens (NA = 0.5).

The main theoretical basis of the computational simulation in this thesis is the Drude model, based on Maxwell’s system of equations for the electromagnetic field simulation of dark-field scattering spectra by the finite element method (FEM) [30]. The FEM involves dividing the spatial region to be analyzed into countless miniature meta-regions, viewing the set of these miniature meta-regions as a continuous whole, and then re-decomposing the partial differential equations of this continuous whole into a set of equations for each meta-region for solving, in order to simplify the complex boundary problem into a finite-degree-of-freedom solution. The main computational software used to perform the finite element method is COMSOL Multiphysics, 6.2. The methodology and the specific parameters of the simulation experiments can be seen in Figure S10 in the Supplementary Information. The optical constants for the metal material within the relevant wavelength range are shown in Table S1.

## 3. Results and Discussion

### 3.1. Construction of Silver Microplate-Molecule-Multi-Sized Silver Nanospheres NPoM Nanocavities

**Figure 1:**
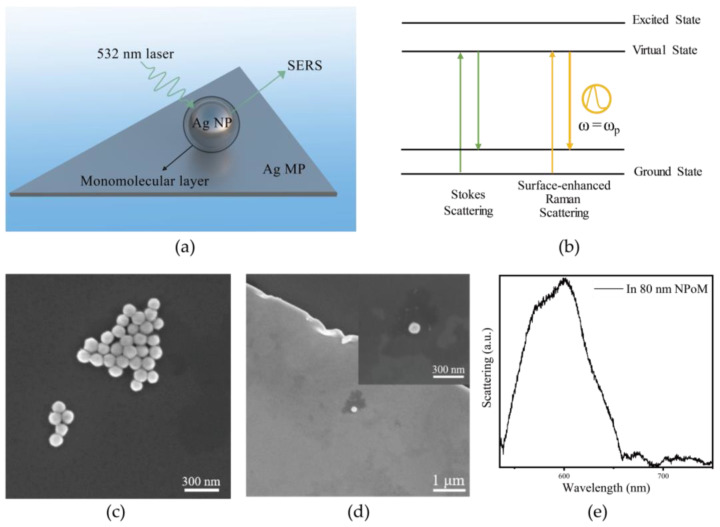
(**a**) Schematic structure of single-crystal silver micrometer sheet–molecule–multi-sized silver nanosphere NPoM nanocavity; (**b**) Mechanism of resonance-enhanced Raman scattering with plasmon; (**c**) The SEM characterization image of the 80 nm silver nanospheres dispersed in deionized water; (**d**) NPoM nanocavities coupled to monomolecular layers; (**e**) Plasmonic resonance peak position of NPoM nanocavity for silver nanosphere size of 80 nm and gap of 1 nm.

In order to achieve the selective and stable enhancement of molecular Raman scattering, we constructed a silver microplate–molecule–multi-size silver nanosphere NPoM nanocavity. As shown in Figure 1a, the NPoM nanocavity was constructed by placing a single layer of molecularly coated silver nanospheres on a silver microplate in a bottom-up manner. The NPoM nanocavity enhances Raman scattering via electromagnetic (EM) effects. When the incident light frequency matches the nanoparticle’s electron resonance frequency, it drives electron movement, increasing the electromagnetic field strength and enhancing molecular polarization, which boosts the Raman scattering intensity (Figure 1b).

To investigate the influence of nanoparticle size on the optical properties of the NPoM system, uniformly sized silver nanospheres with diameters of 60 nm, 80 nm, 100 nm, and 120 nm were synthesized. The characterization diagrams and particle size distribution diagrams of AgNPs with different sizes are shown in Figure S4. The SEM characterization image of the 80 nm silver nanospheres is shown in Figure 1c. NPoM nanocavities with different resonance modes were then constructed on silver microplates. By directly observing the colors in dark-field imaging and comparing the dark-field scattering spectra, nanocavities with appropriate silver nanosphere sizes were selected to match the Raman scattering peaks of different molecules. Specifically, the resonance peaks were well aligned with the Raman scattering positions of 4-MBA and BPDT molecules. The Raman peak positions of these molecules were compared using Raman experimental measurements, with both of these molecules containing benzene rings in their molecular structures. Finally, scanning electron microscope (SEM) characterization experiments were carried out on the selected NPoM nanocavities coupled with molecules, as shown in Figure 1d. This method prevents damage being caused to the nanostructures by the bombardment of high-energy electron beams during electron microscopic characterization. In addition, it also ensures the strength of the interaction between the nanocavities and molecules. Through measurement, it was found that when the silver nanosphere size used to construct the NPoM nanocavity was 80 nm and the gap was 1 nm, the nanocavity exhibited a highly located plasmonic cavity mode with a resonance wavelength of approximately 590 nm, and the plasmonic resonance peak positions matched the 1084 cm^−1^ and 1586 cm^−1^ Raman scattering positions in 4-MBA molecules and BPDT molecules. The dark-field scattering spectra are shown in Figure 1e.

### 3.2. The SERS of 4-MBA Molecules by NPoM Nanocavities

We used the 4-MBA molecule as a probe molecule to examine the SERS performance of NPoM nanocavities and demonstrated the superiority of NPoM nanocavities in realizing the selective and efficient stabilization of SERS. 4-MBA molecules have a large conjugation system, which contains functional groups such as benzene rings and carboxyl groups, and thus their Raman scattering is rich in characteristic peaks. The sulfhydryl group (-SH) in the 4-MBA molecule can be adsorbed on the surface of the silver substrate through the formation of Ag-S bonds [31], which puts the molecule in the SERS-enhanced “hotspot” region [32], which increases the Raman scattering cross-section of the molecule, resulting in a significant enhancement of the Raman scattering. In the hotspot region, the enhancement effect of the electromagnetic field is more significant and can reach a higher intensity. This hotspot effect can greatly improve the sensitivity of SERS, which makes it possible to detect 4-MBA molecules at low concentrations. 4-MBA molecules can not only chemisorb with the metal substrate through the thiol group, but can also chemisorb through the carboxyl group and the metal surface to form hydrogen bonding or electrostatic interactions and other interactions. The synergistic effect of such multiple interactions can further enhance the 4-MBA molecules on the silver substrate.

**Figure 2:**
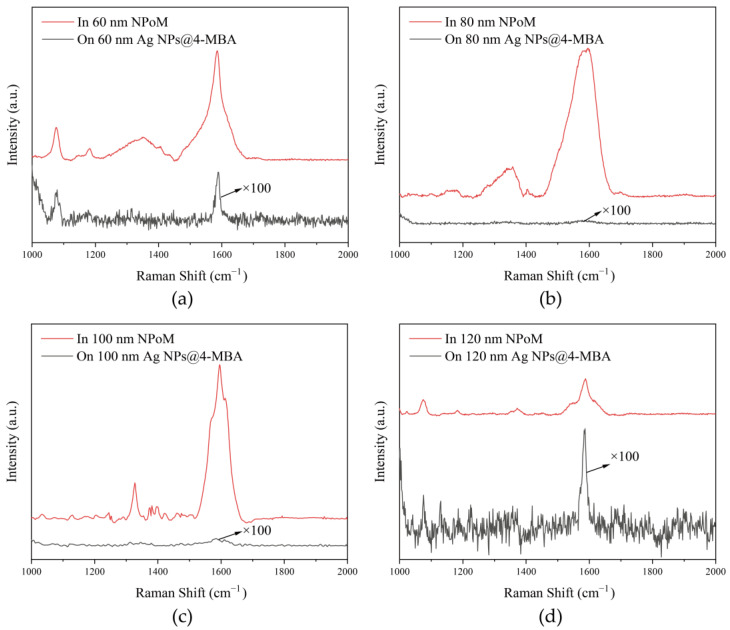
The SERS of 4-MBA molecules on NPoM nanocavities with different sizes. (**a**) 60 nm; (**b**) 80 nm; (**c**) 100 nm; (**d**) 120 nm.

The red Raman scattering lines in Figure 2a–d demonstrate the SERS of the NPoM nanocavity constructed using 60 nm, 80 nm, 100 nm, and 120 nm silver nanospheres for the 4-MBA molecule, respectively. The peak at 1084 cm^−1^ in the figure corresponds to the vibration of the aromatic ring of the 4-MBA molecule; the peak at 1400 cm^−1^ mainly corresponds to the vibration related to the carboxyl group in the 4-MBA molecule; and the peak at 1586 cm^−1^ is the breathing mode of the aromatic ring, which reflects the overall vibration characteristics of the benzene ring and can be used to judge the structural integrity and degree of substitution of the benzene ring [33]. The black Raman scattering lines in Figure 2a–d indicate the Raman scattering of 60 nm, 80 nm, 100 nm, and 120 nm silver spheres wrapped with a single layer of 4-MBA molecules.

The calculation of the SERS enhancement factor involves comparing the SERS signal intensities in two different scenarios: one from the SERS spectrum of a monolayer-covered silver nanoparticle, and the other from the SERS spectrum enhanced by the NPoM nanocavity. The SERS enhancement factor (EF) is obtained by the ratio of these two signal intensities. The formula for the SERS enhancement factor (EF) can be expressed as follows:

(1) $$\mathrm{EF}=\frac{I_{SERS,NPoM}}{I_{SERS,AgNP}}$$

The SERS of the nanocavity is significant compared to the silver nanospheres wrapped with only a single molecule layer. In Figure 2a–d, the Raman scattering at 1084 cm^−1^ was enhanced by 120, 5452, 262, and 69 times, respectively; the Raman scattering within 1250–1450 cm^−1^ was enhanced by 755, 2480, 542, and 28 times, respectively; and the Raman scattering at 1586 cm^−1^ was enhanced by 351, 7904, 2151, and 473 times, respectively. Among them, when the size of the silver nanospheres was 80 nm and the plasmonic resonance peak position matched the Raman shift at 1586 cm^−1^, the SERS enhancement factor was the largest. It can be seen that the vibration of the benzene ring at 1586 cm^−1^ was significantly enhanced by 7904 times.

### 3.3. The SERS of BPDT Molecules by NPoM Nanocavities

In order to further verify the selective Raman scattering enhancement of NPoM nanocavities, we selected BPDT molecules coupled with different sizes of NPoM nanocavities for SERS measurements. The nanocavity is able to localize the electromagnetic field in a small spatial range, making the electromagnetic field strength around the BPDT molecule much higher than that in free space. This localized electromagnetic field is able to interact with the molecule more efficiently and enhance the polarization rate of the molecule, which improves the Raman scattering efficiency and enhances the Raman scattering. Moreover, the BPDT molecule contains a biphenyl structure with a larger conjugation system, which further enhances the SERS [34].

**Figure 3:**
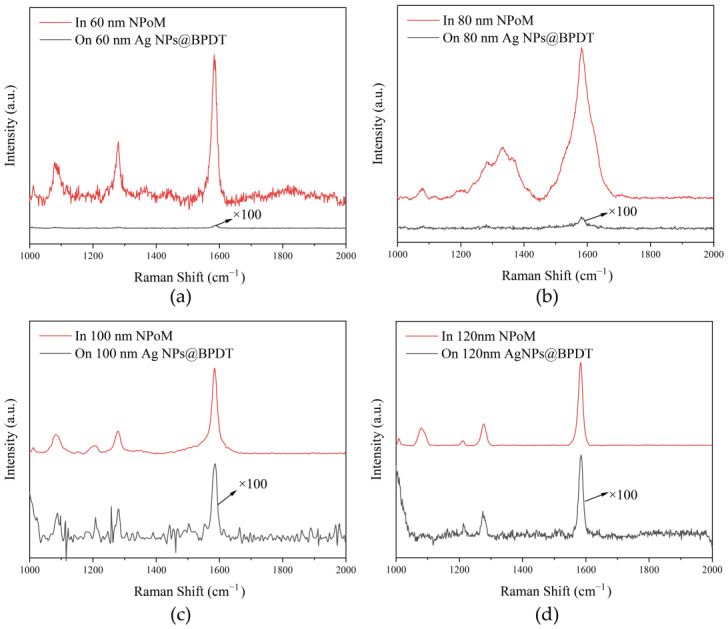
The SERS of BPDT molecules on NPoM nanocavities with different sizes: (**a**) 60 nm; (**b**) 80 nm; (**c**) 100 nm; (**d**) 120 nm.

The red Raman scattering lines in Figure 3a–d demonstrate the SERS of the NPoM nanocavities constructed using 60 nm, 80 nm, 100 nm, and 120 nm silver spheres for the BPDT molecule, respectively. The peak at 1078 cm^−1^ corresponds to the out-of-plane bending vibration of the C-H bond on the benzene ring; the peak at 1278 cm^−1^ corresponds to the coupled vibrational modes of the stretching vibration of the C-C bond and the in-plane bending vibration of the C-H bond of the benzene ring; and the peak at 1580 cm^−1^ corresponds to the backbone vibration of the benzene ring, i.e., the benzene ring has a very strong and stable structure. The peak at 1580 cm^−1^ corresponds to the skeleton vibration of the benzene ring, i.e., the stretching vibration of the carbon–carbon double bond in the benzene ring [35]. The black Raman scattering lines in Figure 3a–d indicate the Raman scattering of 60 nm, 80 nm, 100 nm, and 120 nm silver nanospheres wrapped with a single layer of BPDT molecules. The SERS enhancement of the nanocavity is significant compared to the silver nanospheres wrapped with only a single molecular layer. In Figure 3a–d, the Raman scattering at 1078 cm^−1^ was enhanced by 4745, 840, 109, and 447 times; the Raman scattering at 1278 cm^−1^ was enhanced by 5691, 6781, 122, and 109 times; and the Raman scattering at 1580 cm^−1^ was enhanced by 5055, 1948, 124 and 104 times. The SERS enhancement factor was largest when the silver sphere size was 80 nm and the plasmonic resonance peak position matched the Raman shift, and it can be seen that the vibration of the benzene ring at 1278 cm^−1^ was significantly enhanced by a factor of 6781.

### 3.4. Efficient and Selective SERS with NPoM Nanocavities

**Figure 4:**
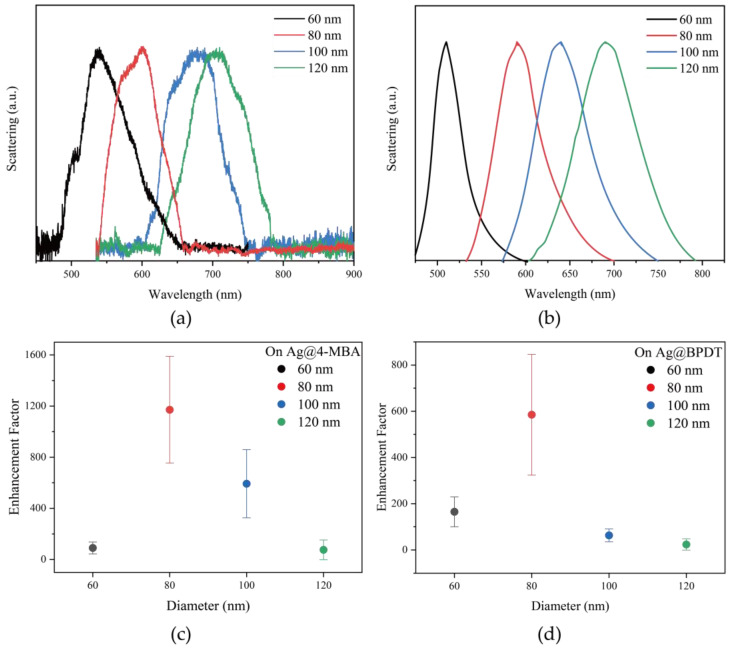
(**a**) Dark-field scattering spectra of AgNPs with different sizes. (**b**) Simulated dark-field scattering spectra of AgNPs with different sizes. Comparison of SERS enhancement factors at 1580 cm^−1^ for nanocavities composed of silver nanospheres with different diameters: (**c**) 4-MBA; (**d**) BPDT.

By analyzing the molecular structures of 4-MBA and BPDT, it can be observed that the 4-MBA molecule consists of a benzene ring, a carboxyl group and a sulfhydryl group, the thickness of the benzene ring is about 0.34 nm–0.37 nm, the sulfhydryl group has a bond length of about 1.34 Å, the length of the C=O bond in the carboxyl group is about 1.23 Å, and the length of the C-O bond is about 1.43 Å. If the molecule is stacked in a relatively tight manner, and the sulfhydryl group and carboxyl group stretch perpendicular to the benzene ring plane, the thickness of the monolayer is about 0.8 nm–1.2 nm. The main body of the BPDT molecule is a biphenyl structure, with a torsion angle between the two benzene rings; the thickness of each benzene ring is about 0.34 nm–0.37 nm, the thickness of the biphenyl portion is about 0.6 nm–0.8 nm, and the length of the S-H bond of the thiol group is about 1.34 Å. The spatial steric hindrance of the biphenyl structure restricts the adsorption orientations of the molecule, and as a result, the gap formed by the nanocavity may be slightly smaller than that for the 4-MBA molecule, leading to a monolayer thickness of approximately 0.9–1.3 nm. We approximate that the thickness of the monomolecular layer for both molecules is around 1 nm. To better compare the effect of different nanoparticle sizes on the enhancement factor, we used atomic layer deposition (ALD) to coat a 1 nm thick Al_2_O_3_ isolation layer between the silver plates and silver spheres. The resonance peak positions corresponding to the silver spheres of different sizes that form the NPoM nanocavities were then measured. As shown in Figure 4a,b, it can be seen that the nanocavity equipartition exciton resonance peaks are red-shifted from 540 nm to 700 nm when the diameter of the silver nanospheres increases from 60 nm to 120 nm. The nanocavity resonance peaks of the silver spheres with a diameter of 80 nm were chosen to better match the Raman shifts of the 4-MBA and BPDT molecules.

In order to test the correctness of this conclusion, we selected NPoM nanocavities constructed with silver nanospheres of different sizes and silver nanospheres coated with 4-MBA molecules and BPDT molecules of different sizes as comparative samples, respectively, and carried out several sets of controlled experiments to count their SERS enhancement factors at 1580 cm^−1^, as shown in Figure 4c,d. It is worth noting that, in a single silver nanoparticle, all the adsorbed molecules contribute to the SERS signal, whereas in NPoM, only the molecules within the nanocavity contribute to the signal. However, in practical measurements, the Raman signal from molecules outside the cavity is weak, so their contribution has a minimal impact when calculating the enhancement factor. The experimental results show that the enhancement factors for both 4-MBA and BPDT molecules are highest when using 80 nm silver nanospheres, with an average enhancement factor reaching 10^3^. When the nanocavity resonance is detuned, the enhancement factor decreases to 10^2^. This indicates that by tuning the resonance peak position of the NPoM nanocavity, one can achieve the selective and efficient stabilization of the SERS signal for a specific molecule.

## 4. Conclusions

In this paper, we focus on the highly efficient detection of SERS for 4-MBA and BPDT molecules using NPoM plasmonic nanocavities, which generates hotspots through localized surface plasmonic resonance between nanoparticles and greatly enhances the Raman scattering of the adsorbed molecules at these hotspots. When the diameter of the silver nanospheres is increased from 60 nm to 120 nm, it is shown that the resonance peak of the NPoM nanocavity shifts from 540 nm to 700 nm (red-shifts), and the resonance frequency of the NPoM nanocavity constructed by the 80 nm silver nanospheres has the best enhancement effect because it matches the Raman scatterings of both the 4-MBA and BPDT molecules. By utilizing the plasmon resonance enhancement effect of the 80 nm NPoM nanocavity, combined with molecular bonding and chemical interactions to enhance intermolecular adsorption, the Raman scattering at 1586 cm^−1^ for the 4-MBA molecule can be enhanced by a factor of 7904 compared to the SERS of the 80 nm silver nanosphere substrate alone. In the resonance Raman enhancement detection of BPDT molecules by NPoM nanocavities, BPDT molecules, which have a larger conjugation system, exhibit a SERS enhancement of up to 6781-fold due to their lower solubility in water. In summary, NPoM nanocavities of different sizes exhibit varying absorption and scattering properties, and their SPR bands have distinct peak wavelengths, which can be adjusted to modulate the optical response, enabling the efficient and stable wide-range detection of various molecules. SERS based on multiscale NPoM nanocavities has multiple advantages and is expected to be applied in biomedical detection and environmental pollutant detection.

## Data Availability

Data is contained within the article or Supplementary Material.

## Supplementary Materials

The following supporting information can be downloaded at: https://www.mdpi.com/article/10.3390/nano15060421/s1, Figure S1: Schematic diagram of the grazing incident dark field scattering spectrum measurement; Figure S2: Schematic diagram of optical path of micro-confocal Raman spectroscopy sister; Figure S3: Scanning Electron Microscope (SEM) images; Figure S4. SEM characterization images and particle size distribution diagrams of silver spheres with different sizes; Figure S5: SERS of 4-MBA molecules on NPoM nanocavities with different sizes; Figure S6: SERS of BPDT molecules on NPoM nanocavities with different sizes; Figure S7. Repeat the experiment on the surface-enhanced Raman scattering (SERS) of 4-MBA molecules by nanocavities of different sizes; Figure S8. Repeat the experiment on the surface-enhanced Raman scattering (SERS) of BPDT molecules using nanocavities with different sizes; Figure S9. (a) the Raman signal from the silicon wafer, (b) the Raman signal from the silver plate alone, (c) the Raman signal from the silver spheres alone, (d) the Raman signal obtained after self-assembling pure silver spheres onto the silver plate; Figure S10. Steps for calculating resonance peak peak positions by finite element analysis in COMSOL Multiphysics; Table S1. The optical constants for the metal material within the relevant wavelength range.

## Abbreviations

The following abbreviations are used in this manuscript:

SERS	Surface-enhanced Raman scattering
4-MBA	4-mercaptobenzoic acid
BPDT	Biphenyl-4,4′-dithiol
EM	Electromagnetic Mechanism
LSPR	Localized Surface Plasmon Resonance
NPoM	Nanoparticle-on-Mirror
MIM	Metal-Insulator-Metal
AgNO_3_	Silver nitrate
AA	Acrylic acid
SEM	Scanning electron microscope
-SH	Sulfhydryl group
