# Supplementary material for: High-Efficiency SERS of 4-Mercaptobenzoic Acid and Biphenyl-4,4′-Dithiol via Nanoparticle-on-Mirror Plasmonic Nanocavities
Source: Nanomaterials (Basel). 2025 Mar 9;15(6):421. doi: 10.3390/nano15060421 (PMC11945916; doi:10.3390/nano15060421)
Supplement: Supplementary file 1 [file nanomaterials-15-00421-s001.zip › nanomaterials-3510496-supplementary.pdf]

nanomaterials

Supplementary Materials

High-Efficiency SERS of 4-Mercaptobenzoic Acid and Biphenyl-4,4′-Dithiol via Nanoparticle-on-Mirror Plasmonic Nanocavities

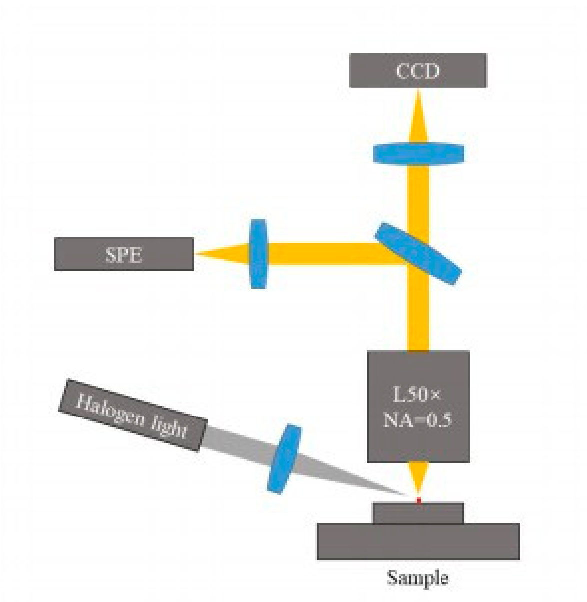

Figure S1. Schematic diagram of the grazing incident dark field scattering spectrum measurement.

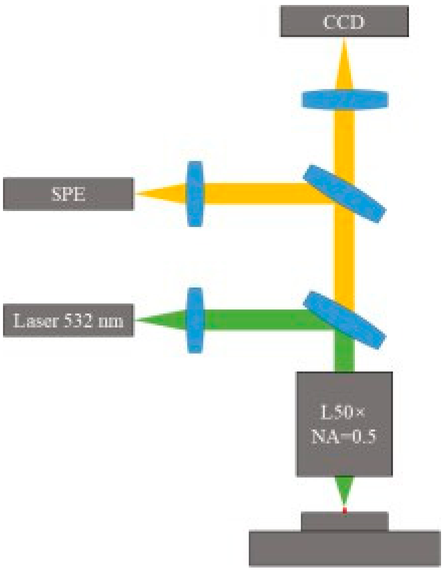

Figure S2. Schematic diagram of optical path of microconfocal Raman spectroscopy system.

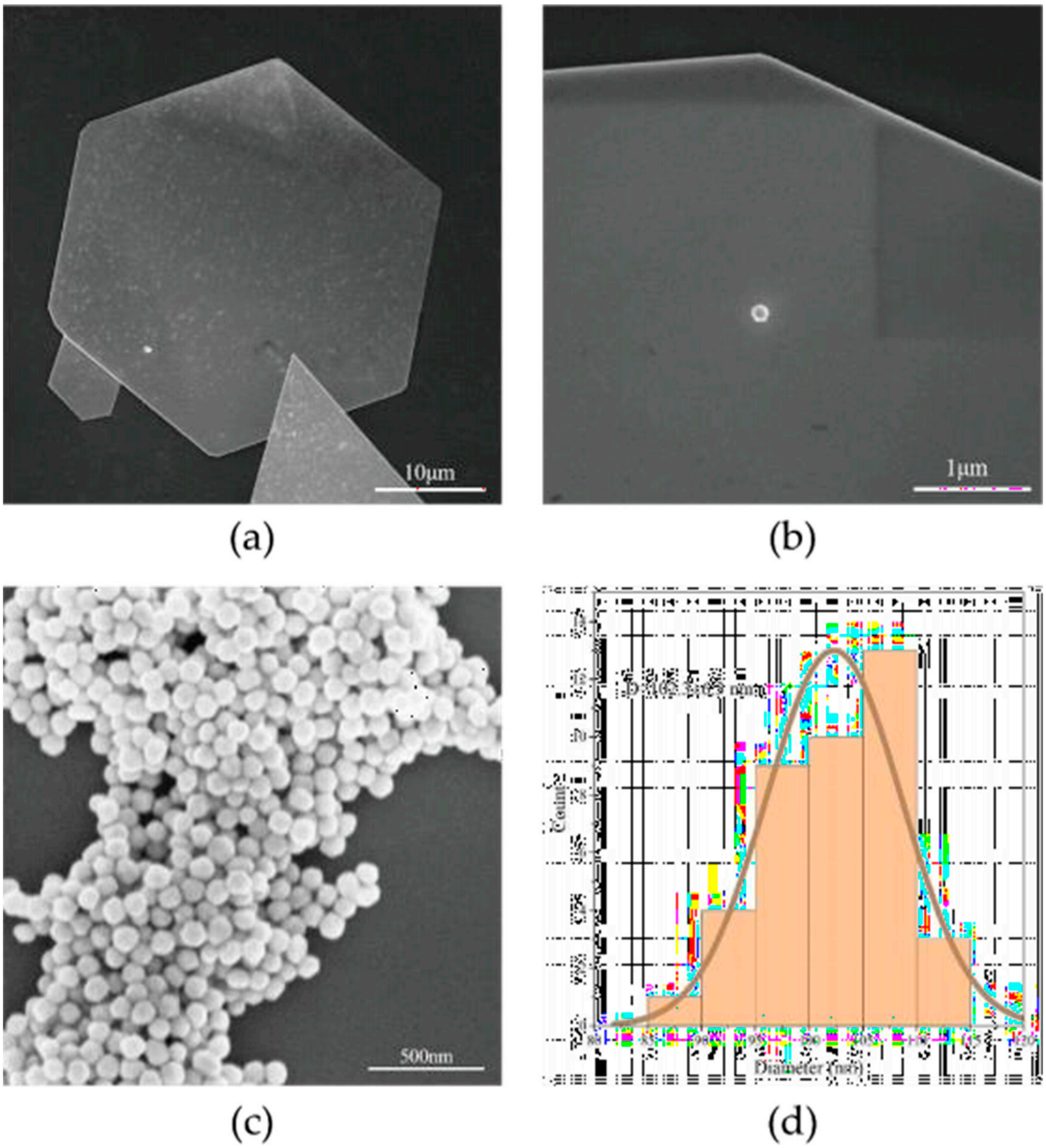

Figure S3. (a) Atomically flat single-crystalline silver microflakes; (b) The NPoM nanocavity composed of 100-nm silver nanospheres is coupled to the monomolecular layer; (c) Characterization diagram of the morphology of 100-nm silver nanospheres; (d) Particle size distribution diagram of 100-nm silver nanospheres.

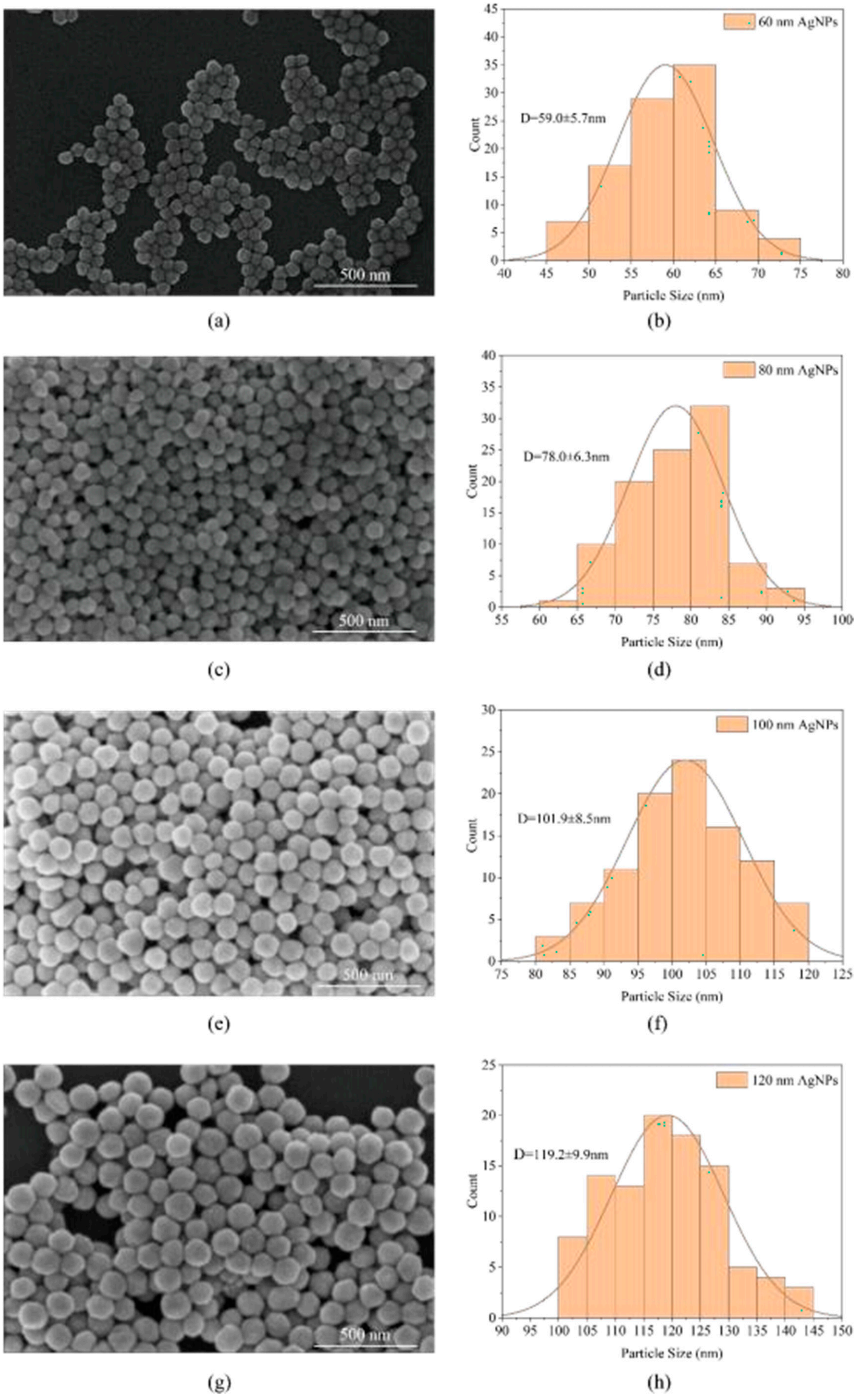
500 nm
60 nm AgNPs
D=59.0±5.7nm
Count
Particle Size (nm)
(a)
(b)
500 nm
80 nm AgNPs
D=78.0±6.3nm
Count
Particle Size (nm)
(c)
(d)
500 nm
100 nm AgNPs
D=101.9±8.5nm
Count
Particle Size (nm)
(e)
(f)
500 nm
120 nm AgNPs
D=119.2±9.9nm
Count
Particle Size (nm)
(g)
(h)

Figure S4. SEM characterization images and particle size distribution diagrams of silver spheres with different sizes. (a) 60nm; (b)80nm; (c) 100nm; (d) 120nm.

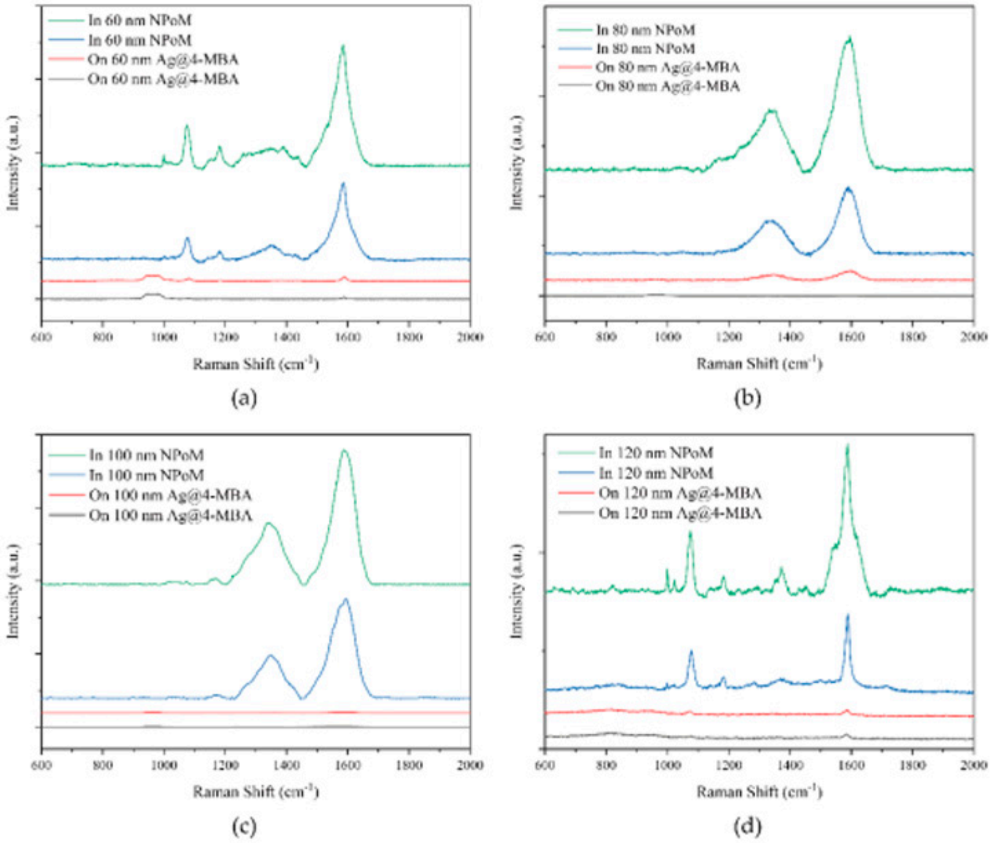

Figure S5. Surface-enhanced Raman scattering (SERS) of 4-MBA molecules on nanoparticle-on-mirror (NPoM) nanocavities with different sizes. (a) 60nm; (b)80nm; (c) 100nm; (d) 120nm.

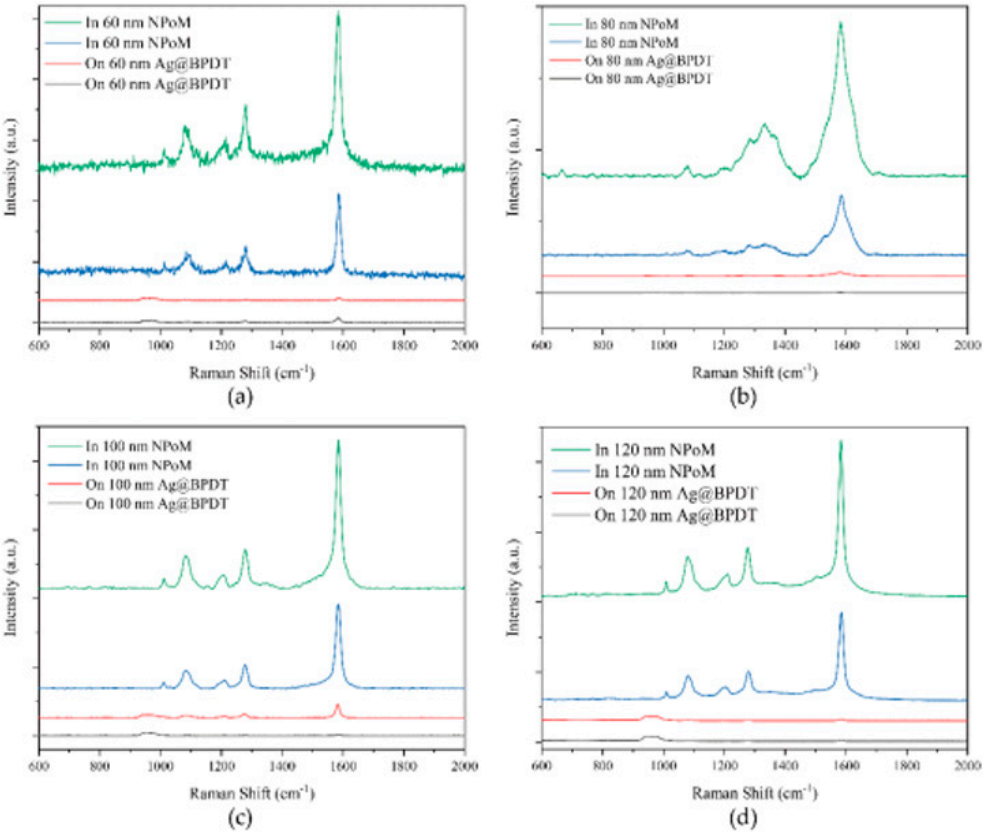

Figure S6. Surface-enhanced Raman scattering (SERS) of BPDT molecules on nanoparticle-on-mirror (NPoM) nanocavities with different sizes. (a) 60nm; (b)80nm; (c) 100nm; (d) 120nm.

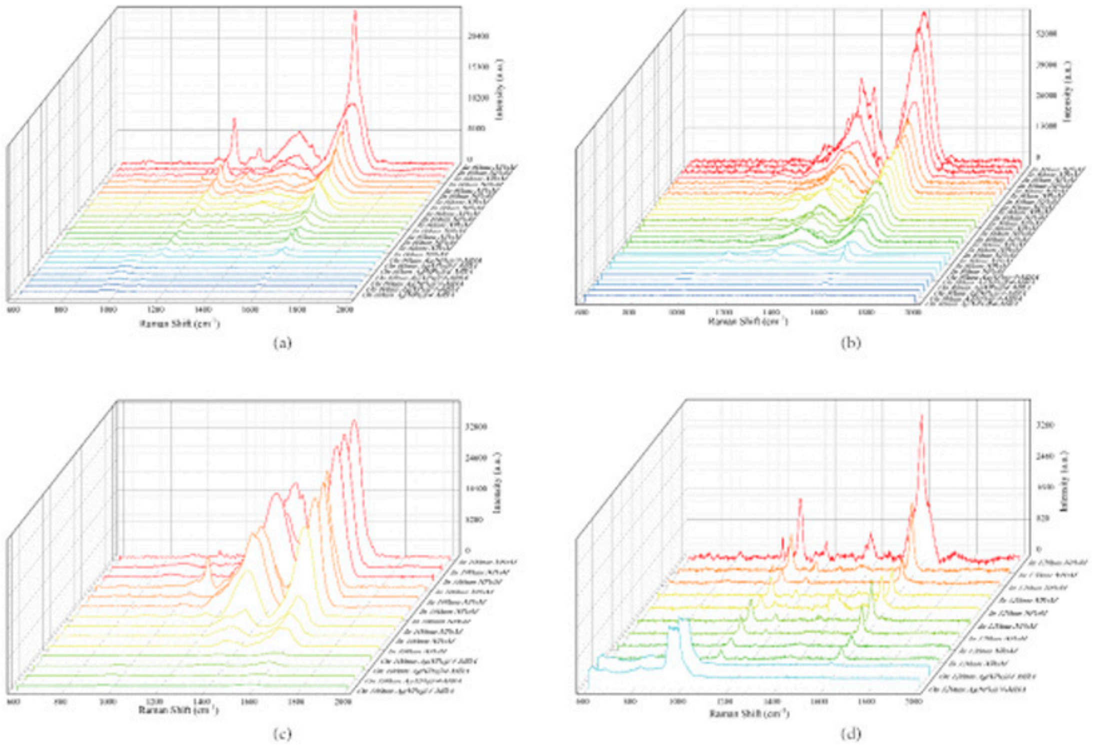

Figure S7. Repeat the experiment on the surface-enhanced Raman scattering (SERS) of 4-MBA molecules by nanocavities of different sizes. (a) 60nm; (b)80nm; (c) 100nm; (d) 120nm.

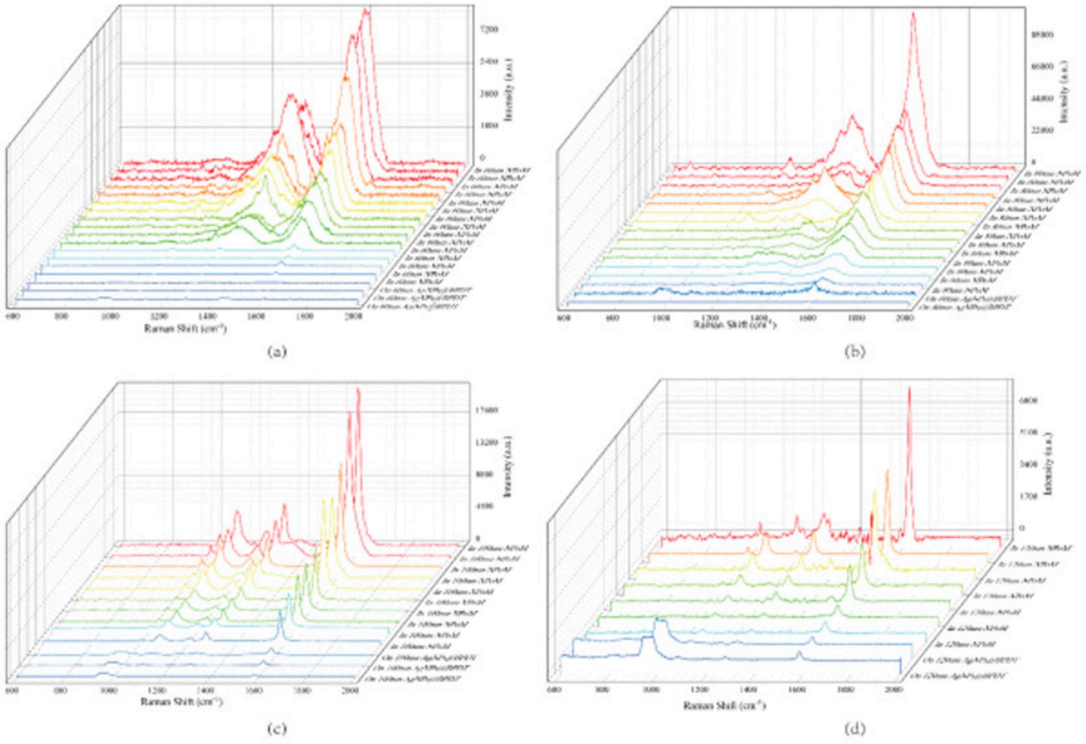

Figure S8. Repeat the experiment on the surface-enhanced Raman scattering (SERS) of BPDT molecules by nanocavities of different sizes. (a) 60nm; (b)80nm; (c) 100nm; (d) 120nm.

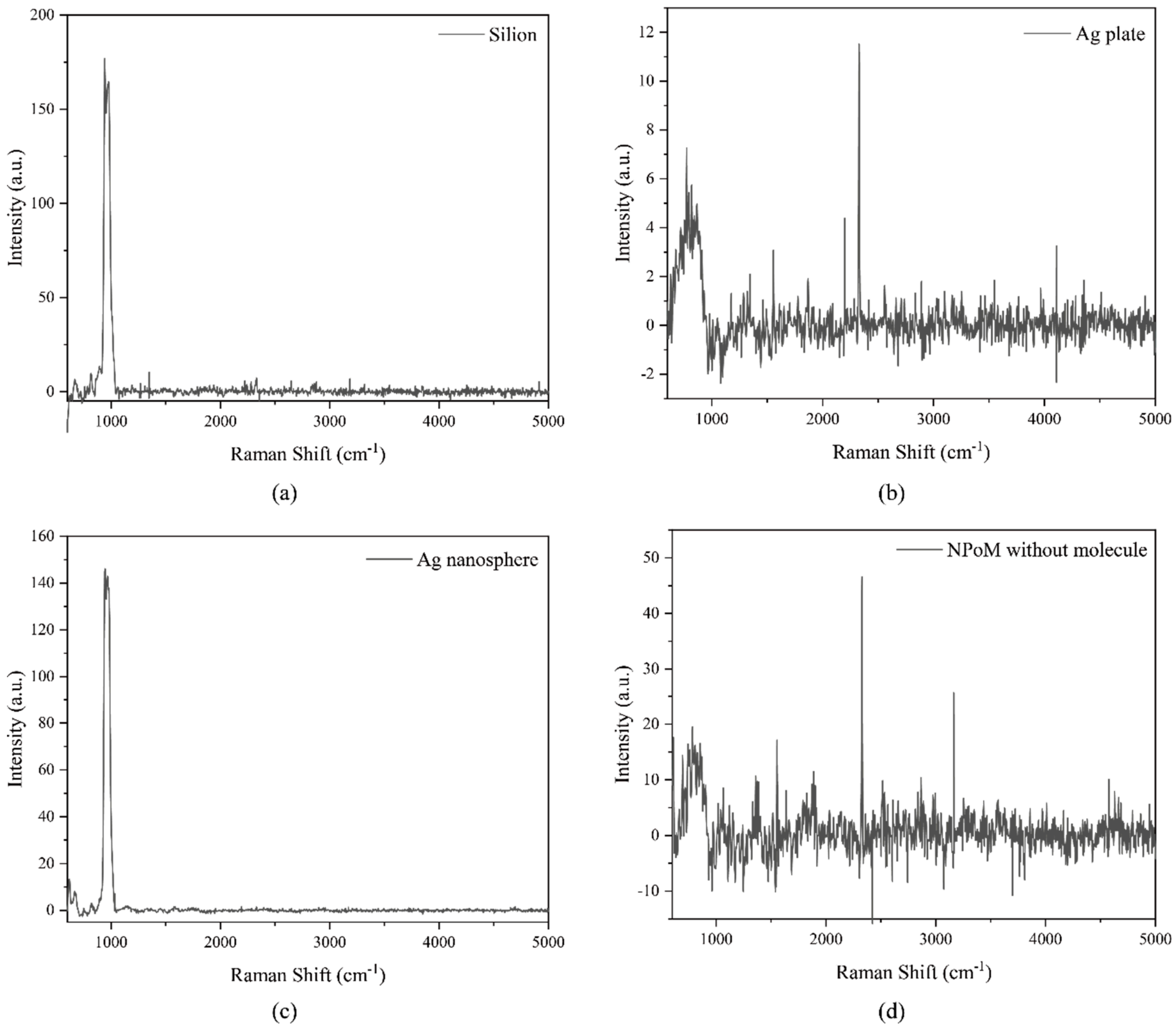

Figure S9. (a) the Raman signal from the silicon wafer, (b) the Raman signal from the silver plate alone, (c) the Raman signal from the silver spheres alone, (d) the Raman signal obtained after self-assembling pure silver spheres onto the silver plate.

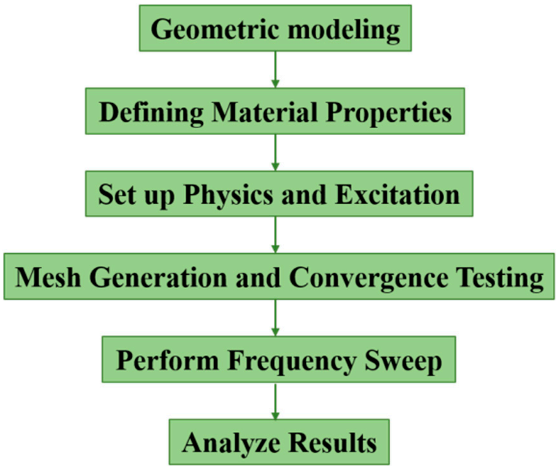

Figure S10. Steps for calculating resonance peak positions by finite element analysis in COMSOL Multiphysics.

Table S1. The optical constants for the metal material within the relevant wavelength range

Element	ε_∞	$\omega_p(eV)$	γ(eV)
Ag[32,62]	4.04	9.172	0.0207
